# Information technology for teaching and learning in a multi-campus public nursing college

**DOI:** 10.4102/hsag.v27i0.1828

**Published:** 2022-05-12

**Authors:** Gopolang Gause, Isaac O. Mokgaola, Mahlasela A. Rakhudu

**Affiliations:** 1 School of Nursing, Faculty of Health Science, North-West University, Mmabatho, South Africa

**Keywords:** nursing, nursing education, public nursing college, teaching and learning, technology usage

## Abstract

**Background:**

Technologies, such as the use of information technology for teaching and learning, e-learning and virtual learning, are commonly used terms in today’s education system. These ever growing and developing modes of teaching and learning have changed the landscape of higher education, in general. As a result, nursing education has equally responded positively to the use of information technology for teaching and learning.

**Aim:**

The aim of this study was to describe and compare the readiness to use information technology for teaching and learning for both nursing students and nurse educators in the two campuses of a North West public nursing college.

**Setting:**

The study was conducted in a multi-campus North West public nursing college in South Africa.

**Methods:**

A quantitative approach of a comparative descriptive design was followed in this study. Descriptive statistics was analysed using the Statistical Package for the Social Sciences (SPSS) Version 27.

**Results:**

A total of 285 (254 nursing students and 31 nurse educators) respondents completed the online questionnaires. Both nurse educators and nursing students were in agreement with the information technology use readiness construct (83.9% and 77.9%, respectively). For all the variables with significant (< 0.05) *p*-values from the Mann–Whitney *U* test, the mean ranks were higher for the Ngaka Modiri Molema District (NMMD) campus.

**Conclusion:**

When comparing the two campuses, conclusion can be drawn that the campus at NMDD is more ready to use information technology for teaching and learning than the campus at Dr Kenneth Kauda District.

**Contribution:**

The results of this study contribute to the body of knowledge on technology use for teaching and learning in nursing education.

## Introduction

Terminologies, such as the use of information technology for teaching and learning, digital, online learning, e-learning and virtual learning, are commonly used in today’s education system. By definition, the use of information technology for teaching and learning can be described as the use of computer-based educational tools and/or systems to conduct the process of teaching and learning (Elbasuony, Gangadharan & Gaber 2018:4). This ever growing and developing model of teaching and learning has changed the landscape for higher education, in general (Yangoz 2017:231). In their study conducted in Iran, Sheikhaboumasoudi et al. (2018:219) revealed that the introduction of information technology in teaching and learning has a potential to improve the learning outcomes. As a result, nursing education has equally responded positively to the use of information technology for teaching and learning as evidenced by its introduction in countries, such as Australia, Canada, United Kingdom and America (Elbasuony et al. 2018:4). However, Hirkani and Supe (2018:77) argue that its use in the category of health professions education seems to be in its infancy stage in some parts of the world, particularly in the underdeveloped states in the African continent.

In an African context, there has been a noticeable increase in technology use and willingness to incorporate its use in almost all sectors of life in countries like Rwanda, with more emphasis placed on the education sector (Harerimana & Mtshali 2020:28). According to a study conducted by Bobtayo, Essel, and Mohammed (2020:11) in Ghana, information technology in nursing education is used for a variety of reasons ranging from academic administration to actually conducting lessons. Furthermore, its use includes lecture preparation for both class activities and assessments (Harerimana et al. 2016:26). Nevertheless, a study conducted by Bobtayo et al. (2020:11) revealed that assessments and record-keeping remain primarily paper-based in most of the nursing and midwifery colleges. This is in line with the anecdotal evidence of the authors of this article being nurse educators and affiliated with the quality assurer body of the public nursing college where this study was conducted. Furthermore, the authors have witnessed limited use of information technology for teaching and learning, including evaluating students’ competence because of issues related to academic integrity, poor connectivity, lack of devices and information technology illiteracy to mention a few.

Despite the above notion, it is inevitable that information technology use is the future of nursing education in order to satisfy the critical cross field outcomes such as lifelong and continuous learning (Pete, Coopasami & Knight 2017:303). The emphasis to the latter became more apparent amidst the coronavirus disease 2019 (COVID-19) era that continues to sweep through the world, to date. This pandemic became a game changer in almost all spheres of life and ushered new ways of working, including nursing habits, nursing practice, and the way in which teaching and learning are approached (Esterhuizen 2020:4; Makumbe 2020:621). The declaration of state of disaster by President Cyril Ramaphosa on 15 March 2020 followed by the introduction of lockdown regulation on 26 March 2020 forced many institutions of learning to adopt virtual teaching and learning modalities. Makumbe (2020:628) raised a question of whether the use of technology in teaching and learning will effectively serve or disadvantage the needy, given the economic disparities that prevail in the country where this study is conducted.

The authors have further observed a dearth of literature on studies related to the phenomenon ‘technology use for teaching and learning at the public nursing colleges in South Africa’. This is despite the fact that public nursing colleges in South Africa produce between 73% and 80% of professional nurses annually (Geyer 2020:27). Although there is willingness to use technology, especially in clinical nursing education, educators feel that they need upskilling with regard to the use of information technology for teaching and learning to bring their A-game (Powell, Scrooby & Van Graan 2020:04; Van Vuuren, Goon & Seekoe 2018:15). Given the above background and the ever-growing use of information technology in nursing education because of the inevitable fourth industrial revolution and the current COVID-19 pandemic, the authors found it befitting to conduct this current study. The researcher intended to conduct this study in an attempt to close the knowledge gape that exists with regard to information technology usage for teaching and learning at the selected two campuses of the North West public nursing college. The aim of this study was to describe and compare the readiness to use information technology for teaching and learning for both nursing students and nurse educators in the two campuses of a North West public nursing college, with the following objectives:

1. To describe the readiness in information technology usage for teaching and learning by nursing students and nurse educators in the two campuses of a North West public nursing college
2. To compare the readiness in information technology usage for teaching and learning by nursing students and nurse educators in the two campuses of a North West public nursing college.

## Research methods and design

### Study design

A quantitative approach of a comparative descriptive design was followed in this study. A quantitative comparative descriptive design is described as a logic of comparison wherein the researcher compares two or more situations or cases which are meaningfully contrasting (Bryman & Bell 2014:114). Similarly in this study, the authors chose comparison descriptive design in order to (1) describe the readiness in information technology usage for teaching and learning by nursing students and nurse educators in the two campuses of a North West public nursing college, and (2) compare the readiness in information technology usage for teaching and learning by nursing students and nurse educators in the two campuses of a North West public nursing college.

### Setting

The study was conducted in the North West province in South Africa. Respondents in the study included the nursing students and nurse educators from a North West Province Multi-campus nursing college. The college consists of two campuses, which are situated in the two districts out of the four districts of the province, namely Dr Kenneth Kaunda District (Dr KKD) and Ngaka Modiri Molema District (NMMD). The two campuses vary with the districts they cater for in terms of student admissions. Dr KKD/Klerksdorp campus predominantly caters for Dr KKD and Bojanala districts, whereas NMMD/Mahikeng campus primarily caters for NMMD and Dr Ruth Segomotsi Mompati Districts. Notably, the Klerksdorp campus is found in a semi-urban area, whilst the Mahikeng campus is in a predominantly rural area, thus leading to disparities concerning structural development and resource allocation. The college offers both pre- and post-registration nursing programmes, which include diploma in nursing and a range of post-graduate diplomas.

### Population and sampling strategy

The study consisted of two sets of population, namely nurse educators and pre-South African Nursing Council (SANC) registration nursing students (level 1–4) enrolled for the academic year 2021 at North West Province Multi-campus nursing college pursuing SANC-R425 or SANC-R171 qualifications. Multi-level stratified random sampling technique was used in this study, given the geographical factors and the fact that data sets did not have common traits (Brink, Van der Walt & Van Rensburg 2018:122). The two campuses were initially divided into two regions known as strata, which was followed by further stratifying students from each campus according to their level of studies which yielded four strata, namely first-, second-, third- and fourth-year nursing students (Table 1). A sample was obtained from each stratum by random sampling (see Table 2).

**TABLE 1:** Population for both nurse educators and nursing students per campus.

Population	*N*
**North West Public Nursing College: Mahikeng campus**	
First-year level	50
Second-year level	76
Third-year level	150
Fourth-year level	58
Nurse educators	20
**North West Public Nursing College: Klerksdorp campus**	
First-year level	50
Second-year level	56
Third-year level	95
Fourth-year level	62
Nurse-educators	20

**TABLE 2:** Sample size for nursing students.

Level of study	Population	Proportion (*n*/*N*)	Sample
**NMMD campus**			
First year	50	50/334 = 0.150	0.150 × 131 = 20
Second year	76	76/334 = 0.228	0.228 × 131 = 30
Third year	150	150/334 = 0.449	0.449 × 131 = 59
Fourth year	58	58/334 = 0.174	0.174 × 131 = 23
**Total**	334	1.000	131
**Dr KKD campus**			
First year	50	50/263 = 0.190	0.190 × 103 = 20
Second year	56	56/263 = 0.213	0.213 × 103 = 22
Third year	95	95/263 = 0.361	0.361 × 103 = 37
Fourth year	62	62/263 = 0.236	0.236 × 103 = 24
**Total**	263	1.000	103

KKD, Kenneth Kaunda District; NMMD, Ngaka Modiri Molema District.

With regard to the nurse educators, the authors used a total sampling technique, where all nurse educators were included in the study because of their small number (*n* = 40). Therefore, the total population for both nurse educators and nursing students at each campus was computed as below.

Nurse educators (*n* = 20), first-year nursing students (*n* = 50), second-year nursing students (*n* = 76), third-year nursing students (*n* = 150) and fourth-year nursing students (*n* = 58), whereas at Klerksdorp campus, it was as follows: nurse educators (*n* = 20), first-year nursing students (*n* = 50), second-year nursing students (*n* = 56), third-year nursing students (*n* = 95) and fourth-year nursing students (*n* = 62). Table 3 depicts the inclusion criteria in this study.

**TABLE 3:** Inclusion criteria and justification.

Inclusion criteria	Justification
**Nurse educators**	
Registered with SANC as a nurse educator. A minimum of 1 year experience as an educator.	Nurse educators are directly involved in teaching and learning To ascertain that such nurse educator understands the teaching and learning processes at the selected institution
**Nursing students**	
Enrolled for 2021 academic year. Pursuing SANC-R425 or SANC-R171 qualifications	To ensure that information received is current For inclusion of all levels, both SANC-R425 and SANC-R171 nursing programmes offered from North West public nursing college are included as they are running parallel because one (SANC-R425) is being phased out, whereas the other one (SANC-R171) is phasing in.

SANC, South African Nursing Council.

To obtain a sample size, the Raosoft sample size calculator was used. An acceptable margin error was set at 5%, with a confidence level of 95% and response distribution at 50%. The sample size (*n* = 234) was determined from a total population of (*n* = 597) nursing students (i.e. 334 from Mahikeng campus and 263 from Klerksdorp campus). For fair representation of the population, the authors further calculated the proportion of students to be selected from each campus as follows: (334/597) × 100 = 55.946% and (263/597) × 100 = 44.054% for the campus at Mahikeng and Klerksdorp, respectively. This then implied that the nursing students sample size for Mahikeng was 0.55946 × 234 = 131 and for Klerksdorp, it was 0.44054 × 234 = 103. The last aspect was to determine the sample size per study level for inclusion in this current study (Table 3).

The selection of students was carried out through a fishbowl technique to obtain the sample at each study level as shown in the above table.

### Data collection

A self-administered questionnaire by Mohammed (2019:6) on ‘E-learning Readiness from Perspectives of Medical Students: A Case Study of University of Fallujah’ was used. The questionnaire had a Cronbach alpha of 0.913 in the original study that was used, which deemed it to be valid and reliable. The data collection tool was piloted with 10% (60 nursing students and four nurse educators) for each of the population, and necessary adjustment was made to suit the context of this study.

The internal consistency specifically to this study was then measured using the Cronbach alpha value within the Statistical Package for the Social Sciences (SPSS) version 27. The amendments of the tool were then made according to the similarity index obtained from the Cronbach alpha report. The authors then considered the value obtained as the Cronbach alpha in this study.

Following the word of mouth recruitment process, the authors requested a permission from the gatekeepers to place the recruitment pamphlet on the notice boards at each campus during block sessions in accordance with their institutional communication policies. The recruitment pamphlet had contact details of authors and independent research assistant, for potential participants to contact the authors and share their preferred method of receiving the link to the questionnaire should they be interested to participate. The questionnaire was then shared with participants who contacted the authors through either WhatsApp or e-mail depending on their preference. The questionnaire comprised six sections, including (1) the informed consent, (2) socio-demographic characteristics, (3) attitude towards the use of technology in teaching and learning, (4) content and culture readiness, (5) technology-use readiness and (6) participation declined.

Respondents were provided up to 14 days to familiarise with the questionnaire, which implied that responses were only obtained after 14 days from the date of sharing the link. That is, no response was received within the initial 14 days as participants were reading through the informed consent, which was in the first section of the questionnaire. Feedback was received from participants electronically through Google docs, which was linked to the authors’ institutional Google drive wherein its password is only known to them, thus ensuring safe data management. Only the authors and statisticians had access to the data.

The key issue involved in data collection was the presence of COVID-19 pandemic, which restricted human interaction and affected the return rate of the questionnaires. In addition, lack of technology skills and devices were key issues as some participants found it either difficult to fill in an online questionnaire or did not fill it in at all. Some questions were returned empty or incomplete because of the aforementioned key issues.

### Data analysis

Descriptive statistics were analysed using the SPSS V27 software. Incomplete questionnaires were not analysed. A descriptive statistical test was used to determine the level of readiness in information technology use by nursing students and nurse educators in the two campuses of a North West public nursing college. Furthermore, the authors ran the Mann-Whitney *U* statistical tests to compare the readiness in technology usage for teaching and learning in the two campuses of a North West public nursing college. For each construct, the Cronbach alpha value was used to ascertain the reliability of the construct (internal consistency).

### Validity and reliability of the data collection tool

This study adapted a data collection tool that was developed by Mohammed (2019:6) on ‘E-learning Readiness from Perspectives of Medical Students: A Case Study of University of Fallujah’, which had a Cronbach alpha of 0.913 and is deemed valid. However, the authors did not rely on the aforementioned Cronbach alpha value because the tool was adapted to suit the current study context. In order to ensure the validity and reliability of the adapted self-administered questionnaire referred to above, the authors conducted a pilot study with a fraction (10%) of the sample size to test if the data collection tool is testing what it is supposed to test, and to check how the units of analysis interpret the questionnaire in the context of this study. Validity and reliability of the data collection tool were ensured by running the Cronbach alpha reliability test. According to Ardian et al. (2018:1515), Cronbach alpha values of 0.6 or greater imply that the reliability of the construct is acceptable. Higher values are even better. The authors adopted this interpretation of the Cronbach alpha value in the current study. As a result, the constructs were reliable, except for content readiness for educators which had a Cronbach alpha value of 0.528. Where the reliability was unacceptable, the items with negative item-correlations were removed to improve the reliability of the construct. According to Tomás-Sábado, Gómez-Benito and Limonero (2005:794), items with negatively corrected item-total correlations jeopardise the reliability of the construct.

### Ethical considerations

An ethical approval (NWU-02071-20-A1) was obtained from both North-West University Health Research Ethics Committee (NWU-HREC) and Provincial Department of Health research, monitoring and evaluation directorate. Subsequent to attaining approval, permission was further sought from individual campus heads who were the gatekeepers at the campuses. Participation of both sets of respondents was voluntary, and the respondents were allowed to withdraw should the need arise. Confidentiality and privacy of information were ensured according to the NWU institutional policy, and are kept for the next five years.

The study used a questionnaire as a data collection tool. According to Brink et al. (2018:139), one of the advantages of using questionnaire is that it ensures anonymity of participants; hence, it contributed in ensuring anonymity of participants. Participants were not required to give out any identifying information in the questionnaire like their names to further ensure anonymity. Both the mediator and the research assistant were required to sign a standard NWU confidentiality form that was binding them to uphold confidentiality at all costs.

Electronic informed consent was obtained voluntarily from the participants without coercion, undue influence or inappropriate incentives. The detailed electronic informed consent was attached to the questionnaire as the initial section and had the ‘do you want to participate in the study’ question. The answer to that question, either ‘yes’ or ‘no’ determined participation to the study. A link to the informed consent that was part of the questionnaire was shared either through WhatsApp or email depending on participants’ preference. In order to ensure an informed consent, potential participants were allowed a maximum of 14 days to peruse through the electronic consent form prior to accepting or declining it.

## Results

The authors adapted a self-administered questionnaire in this study which was used in a case study in the University of Fallujah to assess e-learning readiness by Mohammed (2019:6). The data collection tool used constructs as presented below to describe e-learning readiness in the context of their study. Similar to this study, information technology usage readiness was described and compared using the following constructs as adapted from Mohammed (2019:6): (1) nurse educators and nursing students’ attitude towards the use of information technology in teaching and learning by educators, (2) nurse educators and nursing students’ content and culture readiness regarding information technology use for teaching and learning and c) nurse educators and nursing students’ access to resources and technical skills.

The results are summarised according to the aforementioned socio-demographic and construct descriptions. That is, the authors described the results according to the constructs as the constructs formed sections of the questionnaire that was shared with the participants. Each of the three main constructs in this section was divided into two sub-constructs. The authors firstly describe the summary of the two sub-constructs for each construct for nurse educators followed by the results of nursing students.

### Socio-demographic description

A total of 285 (254 nursing students and 31 nurse educators) respondents completed the questionnaires. For both sets of population, the majority were female respondents (*n* = 228), whereas a minority (*n* = 57) comprised males. About 55.4% of respondents were from NMMD campus which is predominantly rural, and only 42.8% of respondents were from Dr KKD campus which is semi-urban. The majority of respondents were third- (28%) and second-(25%) year nursing students, whereas fourth- and first-year students accounted for a minority (20% and 17%, respectively). Age distribution at first computer use was as follows: below 10 years (8.5%), between 10 and 16 years (17%) and above 16 years (74.4%).

### Description of the constructs

In this section, the authors describe the results by describing the results from the sub-constructs for the two independent populations in this study. The description of the results is separated according to the populations and further presented in pie charts.

#### Construct 1.1: Nurse educators’ attitude towards the use of information technology in teaching and learning

**FIGURE 1:**
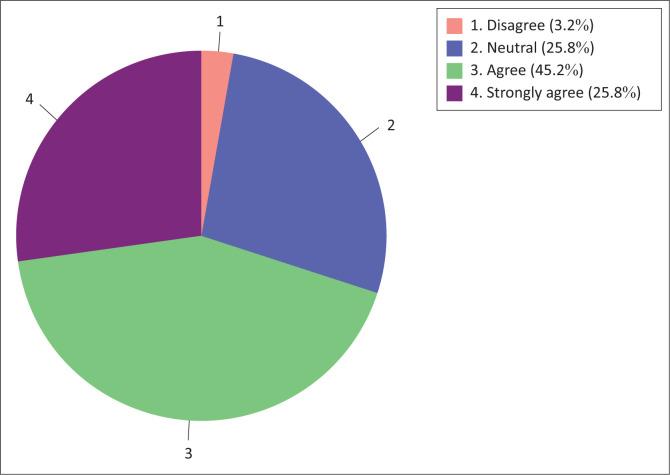
Nurse educators’ attitude towards the use of technology in teaching and learning.

Attitude towards the use of technology in teaching and learning by nurse educators was assessed by the two sub-constructs, namely (1) attitude towards technology use and (2) technology use readiness. The results are presented below in the clustered bar graph (Figure 1). The pie chart shows that without considering the neutral option, the majority (71%) of nurse educators were in agreement (agree to strongly agree) with the construct ‘attitude towards the use of information technology in teaching and learning’ and a minority (3.2%) of nurse educators were in disagreement (disagree to strongly disagree).

**FIGURE 2:**
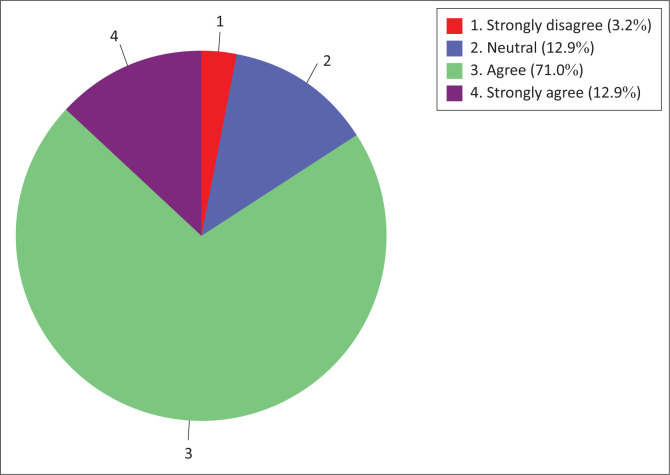
Nurse educators’ technology use readiness.

Similar results were also observed for nurse educators’ ‘technology use readiness’ sub-construct (Figure 2). The majority (83.9%) of nurse educators were in agreement (agree to strongly agree) with the information technology use readiness construct, while a minority (3.2%) of nurse educators were in disagreement (strongly disagree).

#### Construct 1.2: Nursing students’ attitude towards the use of technology in teaching and learning by students and educators

**FIGURE 3:**
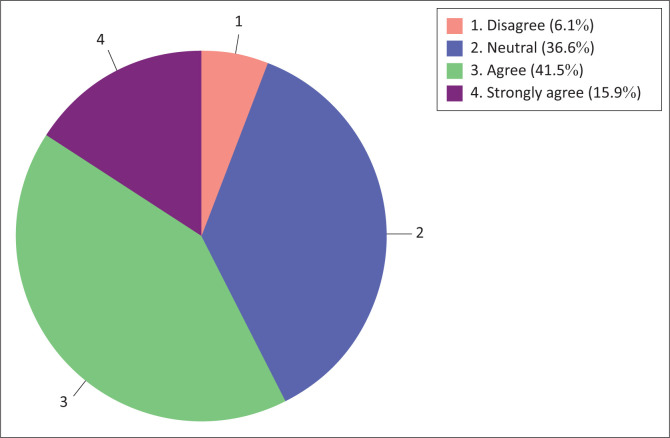
Nursing students’ attitude towards the use of technology in teaching and learning.

The majority (57.4%) of nursing students were in agreement with the sub-construct that described their ‘attitude towards the use of technology in teaching and learning’, whereas a minority (6.1%) of them were in disagreement, see Figure 3.

**FIGURE 4:**
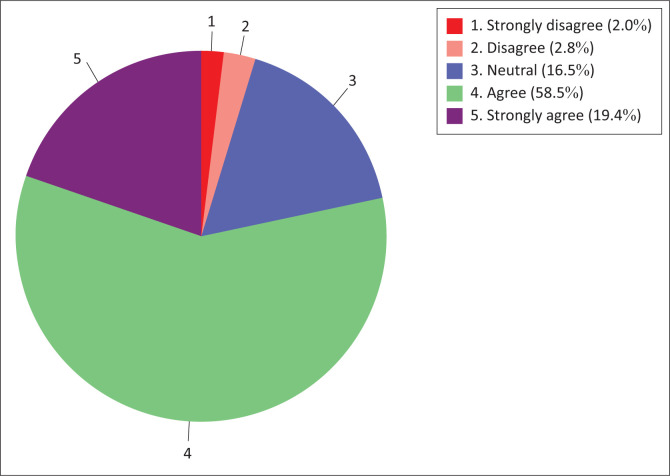
Nursing students’ technology use readiness.

The nursing students’ results (77.9%) further showed a general agreement (agree to strongly agree) to information technology use readiness sub-construct, while those in disagreement were a minority (4.8%). The results of this sub-construct are summarised in Figure 4.

#### Construct 2.1: Nurse educators’ content and culture readiness regarding the use of information technology for teaching and learning

**FIGURE 5:**
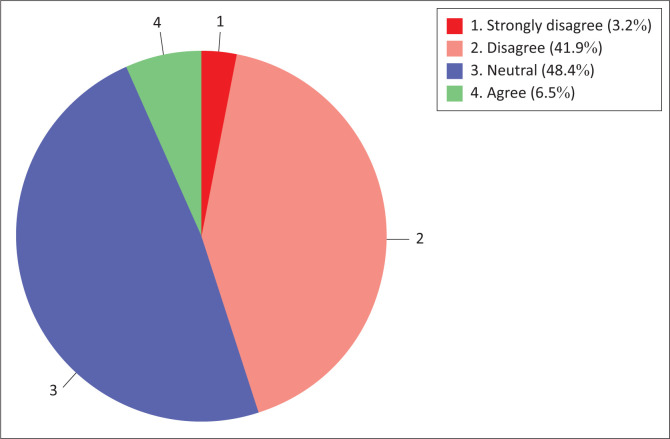
Nurse educators’ content readiness.

The results of this study revealed that the majority (45.1%) of nurse educators are in disagreement with the content readiness regarding the use of information technology for teaching and learning sub-construct (Figure 5). A minority (6.5%) of respondents were in agreement. Notably, nearly half (48.4%) of the respondents neither agreed nor disagreed with the nurse educators’ content and culture readiness sub-construct.

**FIGURE 6:**
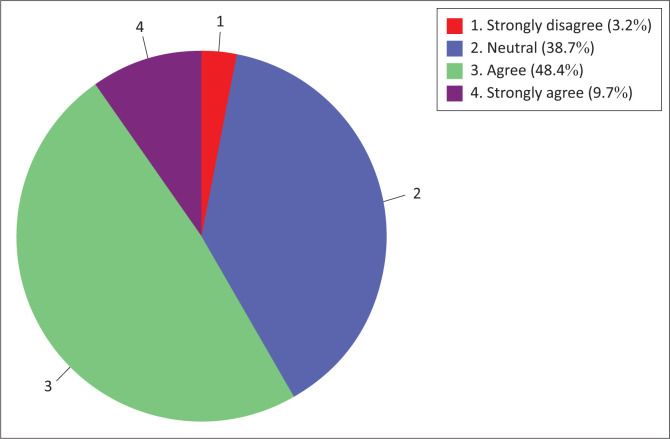
Nurse educators’ culture readiness.

The different results were observed for culture readiness sub-construct as depicted in Figure 6. The majority (58.1%) of nurse educators were in agreement (agree to strongly agree) with the items that described their culture readiness to use information technology for teaching and learning, whereas a minority (3.2%) of them were in disagreement (strongly disagree). A relatively high percentage (38.7%) of nurse educators were also neither in agreement nor in disagreement.

**FIGURE 7:**
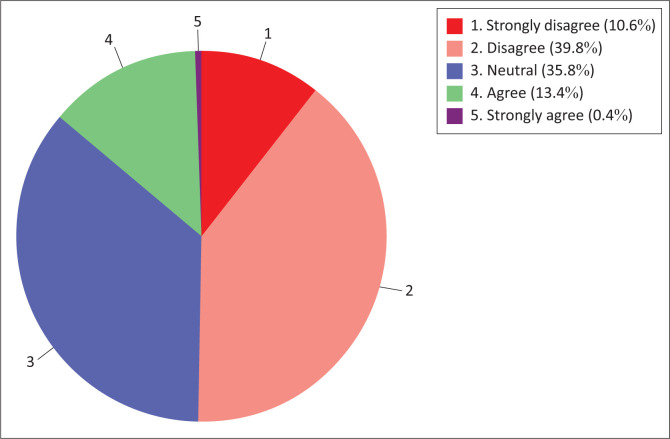
Nursing students’ content readiness.

More than half (50.4%) of the nursing students were in disagreement (disagree to strongly disagree) with the content readiness regarding information technology use for teaching and learning sub-construct. Only a few (13.4%) respondents were in disagreement (agree to strongly agree), see Figure 7.

**FIGURE 8:**
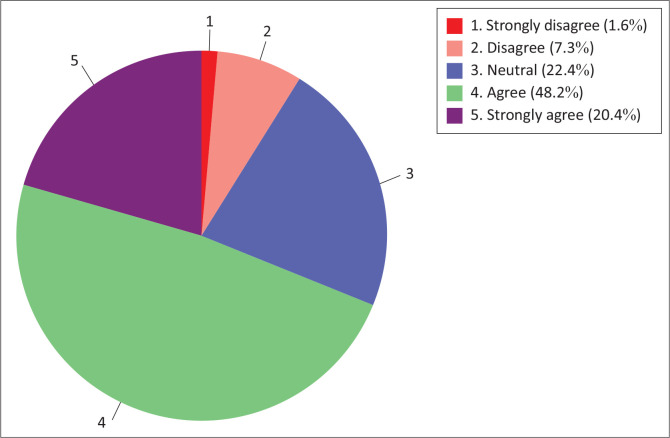
Nursing students’ culture readiness

Different results were observed with regard to culture readiness to use information technology for teaching and learning sub-construct (Figure 8). About 68.6% of respondents were in agreement (agree to strongly agree) with the culture readiness sub-construct, whereas a minority (8.9%) of the respondents were in disagreement (disagree to strongly disagree).

#### Construct 3.1: Nurse educators’ access to resources and technical skills

**FIGURE 9:**
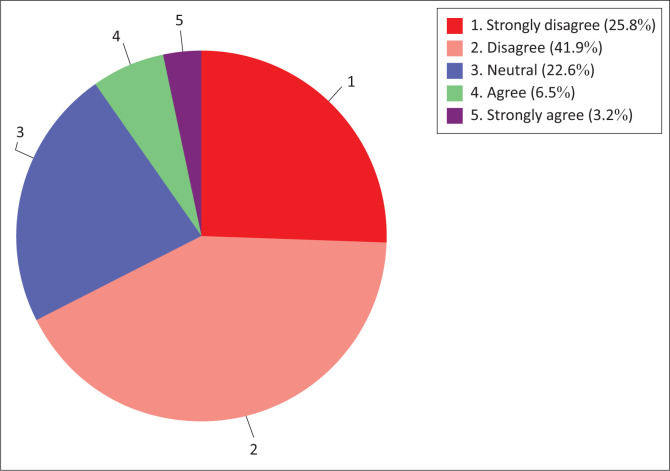
Nurse educators’ access to resources.

**FIGURE 10:**
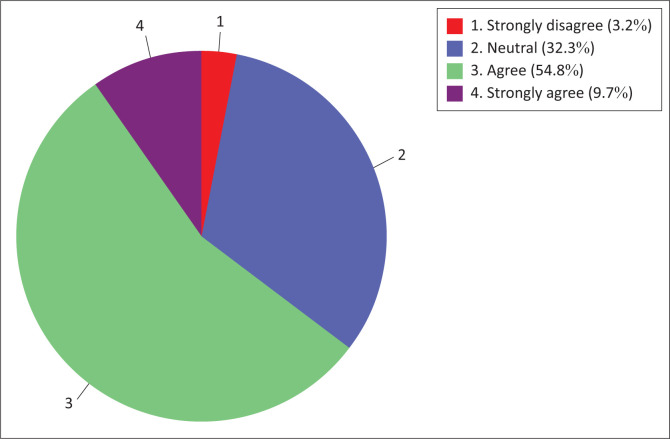
Nurse educators’ technical skills.

This construct aimed at assessing information technology use readiness of nurse educators by assessing their access to resources and technical skills (Figure 9 and Figure 10). The results showed that the majority (67.7%) of nurse educators were in disagreement (disagree to strongly disagree) with the sub-construct ‘access to resources’ that can enable them to use information technology for teaching and learning. A minority (9.7%) were in agreement (agree to strongly agree).

The nurse educators’ results on the sub-construct ‘technical skills’ showed that the majority (64.5%) of respondents were in agreement (agree to strongly agree) with the sub-construct that described the technical skills they possessed to enable them to use information technology for teaching and learning. A minority (3.2%) of respondents were in disagreement (disagree to strongly disagree) with the items in this sub-construct.

#### Construct 3.2: Nursing students’ access to resources and technical skills

**FIGURE 11:**
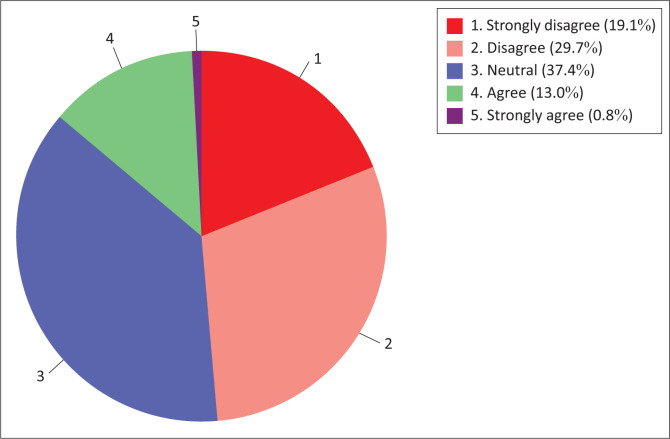
Nursing students’ access to resources.

The results of this study revealed that without considering the neutral option, the majority (48.8%) of nursing students were in disagreement (disagree to strongly agree) with the sub-construct ‘access to resources’ needed to use information technology for teaching and learning. A minority (13.8%) of respondents were in agreement (agree to strongly agree) with the sub-construct of nursing students access to resources (see Figure 11).

**FIGURE 12:**
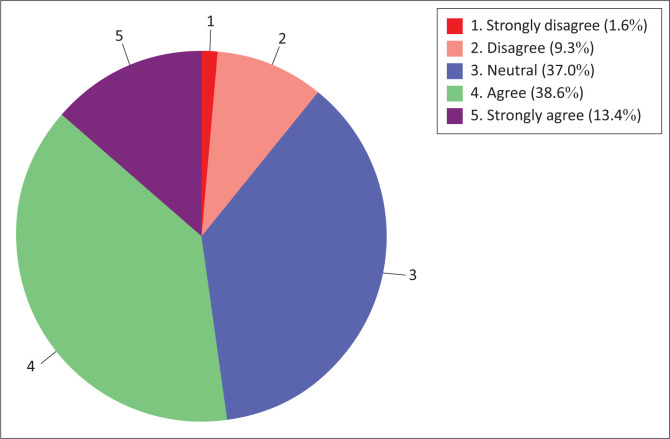
Nursing students’ technical skills.

The difference was observed with the sub-construct that described the nursing students’ technical skills (Figure 12). The majority (52%) of the respondents were in agreement (agree to strongly agree), whereas a minority (11%) of the respondents were in disagreement (disagree to strongly disagree).

### Comparison of readiness in information technology usage for teaching and learning in the two campuses of a North West public nursing college

The authors did not run the comparative statistics for nurse educators because of their small sample size. As a result, there was no report on the comparison of readiness in information technology usage for teaching and learning for nurse educators. The authors acknowledge that the aforementioned was a limitation to the study. Comparative statistics for nursing students on both campuses were run on the following variables: (1) attitude towards information technology usage in teaching and learning, (2) attitude towards information technology usage readiness, (3) content readiness, (4) culture readiness, (5) access to resources and (6) technical skills.

The results of this study revealed that the students’ attitude towards information technology usage in teaching and learning, attitude towards the use of information technology readiness, culture readiness, access to resources and technical skills differ significantly across campuses because the *p*-values for the Mann-Whitney *U* test for these variables are significant at 5% level of significance. However, students’ content readiness does not differ across the campuses because the *p*-values for the Mann-Whitney *U* test for these variables are insignificant at 5% level of significance (Table 3).

Table 4 further explains the difference of the Mann-Whitney *U* test observed in Table 3.

**TABLE 4:** Comparative statistics for campuses.

Variables	Test statistics	
	Mann-Whitney *U*	*p*
Attitude towards the use of information technology in teaching and learning	90.000	0.205
Attitude towards the use of information technology readiness	99.500	0.311
Content readiness	105.000	0.511
Culture readiness	96.000	0.297
Access to resources	107.500	0.602
Technical skills	95.500	0.279

Grouping variable: Campus.

## Discussion

The aim of this study was to describe and compare the readiness in information technology usage for teaching and learning for both nursing students and nurse educators in the two campuses of a North West public nursing college. The discussion of the results will be according to the set of objectives.

### The level of readiness in information technology usage by students and educators in a selected public nursing college

The results of this study revealed that both educators and students are not yet ready to use information technology for teaching and learning. Nurse educators reported not being content ready and not having access to resources that can enable them to use information technology for teaching and learning. Content readiness and access to resources are key prerequisites for the use of information technology for teaching and learning. Lack thereof can be regarded as hindrances for optimum use of information technology for teaching and learning. Similar results were reported by Harerimana et al. (2016:88) in their study in Rwanda, wherein they highlighted the lack of information technology resources as a challenge facing optimum use of technology at nursing education institutions. This was despite their results having showed a generally positive attitude towards the use of information technology for teaching and learning. Pete et al. (2017:302) describe the prerequisites for the use of technology in teaching and learning as (1) psychological readiness, (2) technological readiness and (3) equipment readiness. In this study, nurse educators proved to be psychologically ready because of their positive attitude; however, technological readiness and equipment readiness were lacking.

A similar trend was observed with nursing students in this study. Nursing students showed a positive attitude regarding the use of information technology for teaching and learning. This implies that nursing students are psychologically ready to adopt information technology in teaching and learning. Despite the psychological readiness, there is still a challenge with regard to content readiness. There was a disagreement in the construct of content readiness which inferred that nursing students are not content ready to adopt information technology as a teaching and learning strategy. Equally important was the finding that showed that access to resources still remains a hindrance for the use of information technology for teaching and learning. These findings are supported by a study conducted by Nsouli and Vlachopoulos (2021:14), wherein they established that lack of technology infrastructure and lack of technology apparatus were some of the challenges facing the optimum use of technology for teaching and learning in their context. According to Lee, Yeung and Ip (2017:105) desire for learning which can be translated to positive attitude was found to be associated with computer use for teaching and learning. However, according to Singh (2020:26), online pedagogy is dependent on faculties and students’ readiness, the attitude and skills, and knowledge to use technology for teaching and learning. Therefore, it can be concluded that both students and nurse educators at a North West public nursing college are not ready to use technology for teaching and learning.

### Comparison of readiness in technology usage for teaching and learning in the two campuses of a North West public nursing college

The authors used the Mann-Whitney *U* test to compare the readiness in information technology usage for teaching and learning in the two campuses of a North West public nursing college. The Mann-Whitney *U* test is used to compare differences between two independent groups when the dependent variable is either ordinal or continuous but not normally distributed. In this study, the variables are assumed to be ordinal because their categories are ordered from strongly disagree to strongly agree. For all the variables with significant *p*-values (< 0.05) from the Mann-Whitney *U* test, the mean ranks are higher for the NMMD campus as displayed in Table 5. As the higher values (4 and 5) represent agree and strongly agree, higher mean ranks imply that most of the students from the NMMD campus were more in agreement (agree to strongly agree) with the sentiments in the questionnaire than those from the Dr KKD Campus. As a result, the comparative statistics showed that the NMMD campus is more ready to use technology for teaching and learning than the Dr KKD campus.

**TABLE 5:** Ranks per campus.

Variables	Campus	*N*	Mean rank	Sum of ranks
Attitude towards information technology usage in teaching and learning	Dr KKD Campus	103	112.17	11554.00
	NMMD Campus	141	130.04	18336.00
	Total	244	-	-
Attitude towards the use of information technology readiness	Dr KKD Campus	104	110.16	11457.00
	NMMD Campus	141	132.47	18678.00
	Total	245	-	-
Content readiness	Dr KKD Campus	103	117.37	12089.00
	NMMD Campus	141	126.25	17801.00
	Total	244	-	-
Culture readiness	Dr KKD Campus	103	111.70	11505.00
	NMMD Campus	140	129.58	18141.00
	Total	243	-	-
Access to resources	Dr KKD Campus	104	134.46	13983.50
	NMMD Campus	140	113.62	15906.50
	Total	244	-	-
Technical skills	Dr KKD Campus	104	107.43	11173.00
	NMMD Campus	140	133.69	18717.00
	Total	244	-	-

KKD, Kenneth Kaunda District; NMMD, Ngaka Modiri Molema District.

## Conclusion

The results of this study show that there are gaps as far as readiness in information technology usage for teaching and learning is concerned at the selected public nursing college’s two campuses. Although many respondents (71% and 57.4% for nurse educators and nursing students, respectively) reported a positive attitude to adopt information technology for teaching and learning, much still needs to be done in terms of skill and resource empowerment. As a result, it can be concluded that the North West public college campuses are not ready to use information technology for teaching and learning. However, when comparing the two campuses, conclusion can be drawn that the campus at NMDD, apart from the aforementioned hindrances, is more ready to use technology for teaching and learning than the campus at Dr KKD.

### Recommendations

The authors recommend further research on information technology usage for teaching and learning at public nursing colleges. Furthermore, the authors recommend that further research on attitudes of male nursing students towards the nursing profession needs to be conducted because of their low turnover in this study as it became apparent in the demographics, wherein the majority (*n* = 228) of them were female students, whereas male students accounted for a minority (*n* = 57). In conclusion, it is recommended that the North West department of health capacitates both nurse educators and students at public nursing colleges with the necessary technological skills and equipment needed for use in teaching and learning. These recommendations to upskill students and their educators are made based on the results of this study; the status quo as a result of COVID-19, the fast approaching fourth industrial revolution and the technologically inclined generations that are currently admitted at institutions of higher learning.
